# Association of Psoas: Lumbar Vertebral Index (PLVI) with Postherpetic Neuralgia in Patients Aged 60 and Older with Herpes Zoster

**DOI:** 10.3390/jcm13113100

**Published:** 2024-05-25

**Authors:** Sang-Mee An, Ji Seon Chae, Hyun Jung Lee, Sooyoung Cho, Jiwoong Im

**Affiliations:** 1 Department of Anesthesiology and Pain Medicine, Ewha Womans University Seoul Hospital, Seoul 07804, Republic of Koreaanlhj@ewha.ac.kr (H.J.L.); 2 Department of Anesthesiology and Pain Medicine, College of Medicine, Ewha Womans University, Seoul 07804, Republic of Korea; 3 Department of Anesthesiology and Pain Medicine, Ewha Womans University Mokdong Hospital, Seoul 07985, Republic of Korea

**Keywords:** sarcopenia, older adults, psoas: lumbar vertebral index, frailty, herpes zoster, postherpetic neuralgia

## Abstract

**Background/Objectives**: The psoas: lumbar vertebral index (PLVI) is a simple and convenient measure to assess central sarcopenia. Recent studies have utilized the psoas area to indirectly assess sarcopenia and frailty, exploring their associations with various health outcomes. This study aims to investigate the relationship between the PLVI and postherpetic neuralgia (PHN) in patients aged 60 years and above following a herpes zoster (HZ) infection. **Methods:** We conducted a retrospective analysis of data from 351 patients (≥60 years) who developed HZ between January 2019 and December 2023; the patients were divided into two groups based on the presence or absence of PHN after HZ onset. **Results:** The analyses using receiver operating characteristic curves revealed a value for the area under the curve of 0.813 for PLVI and 0.769 for the modified frailty index (mFI). In a multivariate logistic regression analysis, numerical rating scale scoring, a low PLVI, and a greater number of categorical mFI variables (adjusted odds ratio: 1.30, 3.27, and 2.46, respectively) were found to be significant independent predictors of PHN. **Conclusions:** Our findings highlight the association between a low PLVI and PHN in an older population. The PLVI may have potential as a predictive tool for PHN in older patients with HZ, but further research is needed to confirm these results.

## 1. Introduction

Postherpetic neuralgia (PHN) is the most common complication after infection with herpes zoster (HZ). HZ is caused by reactivation of the varicella-zoster virus, which remains dormant in the dorsal root ganglia following a primary varicella infection. Reactivation typically occurs in older adults or individuals with compromised immune systems, resulting in a painful rash that can lead to PHN [1,2,3,4]. PHN can evolve into a serious and incapacitating condition, affecting every facet of an individual’s life and imposing considerable economic and social strain on caregivers [5,6]. Factors that are independently associated with the emergence of PHN include age older than 60 years, female sex, intensity of acute pain at onset, and duration of rash before confirmation of the diagnosis of HZ infection [6]. Additionally, patients with significant immunodeficiencies or diseases that compromise the immune system, such as diabetes mellitus, face heightened risks. The likelihood of developing PHN markedly escalates when multiple adverse risk factors coexist [1,7] and the incidence of PHN escalates with advancing age [5,6,8].

Frailty, an age-associated condition marked by increased vulnerability and a decline in physiological functions, further exacerbates the risk of developing PHN [8,9,10,11,12]. Frailty is marked by signs such as unintentional weight loss, expressed fatigue, muscle weakness, reduced walking speed, and diminished levels of physical activity [9,10]. Within the broad scope of research on this topic, the influence of aging on the immune–endocrine axis and its contribution to frailty are key areas of interest [9,11]. The development of immunosenescence increases the risk of contracting infectious diseases [12]. Population aging is a global trend with profound and diverse effects on societies. The World Health Organization (WHO) reported that, as of 2020, approximately 962 million individuals worldwide were 60 years or older. This figure is expected to rise to 2.1 billion by 2050, indicating a notable demographic transition toward an older population [13]. With the aging global population, the incidence of HZ and subsequent establishment of PHN are anticipated to rise among frail older adults, posing major challenges in terms of management, enduring complications, and preventive measures [8]. 

Sarcopenia, recognized as a prominent aspect of frailty, is characterized by a gradual reduction in muscle mass and strength [9,14]. Evaluation of the cross-sectional area of the psoas muscle on computed tomography (CT) is an established technique for determining sarcopenia [15,16,17,18]. Considering the diminishing physical and cognitive capabilities of older individuals, accurately gauging frailty remains a significant challenge; the psoas: lumbar vertebral index (PLVI) has been identified as a viable alternative for evaluating frailty levels [16,18]. 

The pathogenesis of PHN remains incompletely understood [3,19]; however, considerable research efforts are directed towards identifying risk factors that predispose individuals to PHN [1,6,7]. Understanding these risk factors is essential for implementing proactive treatments in vulnerable patient populations to prevent its onset [3,19]. Advanced age, a known risk factor for PHN, is intrinsically linked with frailty, and several studies have demonstrated a significant association between PHN and decreased levels of physical activity [1,2,3,4,8]. This led us to hypothesize a potential relationship between PHN and sarcopenia, an essential component of the frailty phenotype representing physical activity.

While several studies have explored the relationship between psoas muscle area and various clinical outcomes [15,16,17,18,20,21,22], the association between a reduced PLVI and the incidence of PHN has not been thoroughly investigated. We hypothesized that PLVI, calculated from CT scans, along with frailty scores, effectively predict the risk of PHN in individuals aged 60 years and older who have been affected by HZ.

## 2. Materials and Methods

### 2.1. Study Design and Data Collection

This retrospective analysis was approved by the Ethical Review Board of Ewha Womans University Seoul Hospital (SEUMC 2024-04-016), and the requirement for written informed consent from the participants was waived. The data were retrieved from the electronic health records of older patients diagnosed with herpes zoster (HZ) infection across a 5-year period (January 2019–December 2023) at the same institution.

Records were considered for inclusion if: (1) the diagnosis of HZ was within one month at the pain clinic outpatient department; (2) the patient was 60 years or older; and (3) either magnetic resonance imaging (MRI) or computed tomography (CT) scans were available that adequately showed the area of the psoas muscle and the vertebral body at the L4 level.

**Figure 1:**
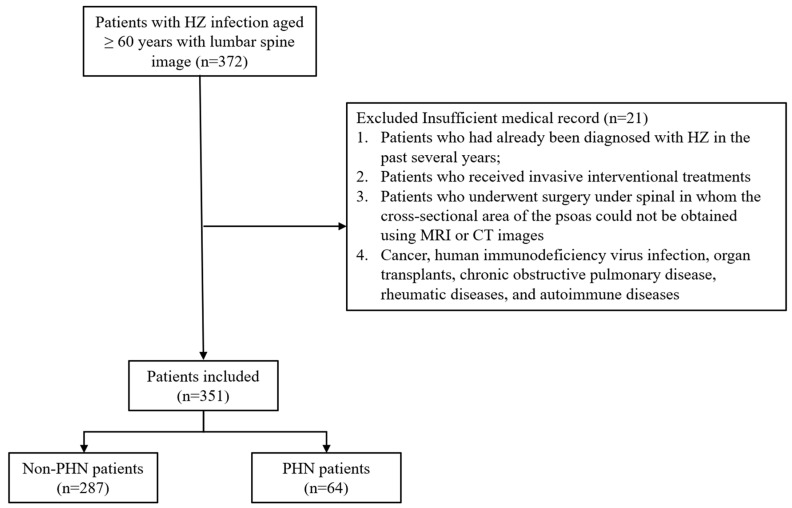
Study flowchart. CT, computed tomography; HZ, herpes zoster; MRI, magnetic resonance imaging; PHN, postherpetic neuralgia.

The exclusion criteria were as follows: (1) past diagnoses of HZ infection (in recent years); (2) records of radiofrequency ablation for alleviating zoster-associated pain or other invasive interventional treatments; (3) spinal surgeries—multiple-fracture surgeries that did not allow accurate measurement of the cross-sectional area of the psoas and vertebral body on CT due to the presence of hardware in the lumbar area; or (4) diagnostic records of cancer, HIV infection, organ transplants, chronic obstructive pulmonary disease, or rheumatic and autoimmune diseases, as these conditions or their treatments may lead to immunosuppression, complicating the correlation between HZ and postherpetic neuralgia (PHN) [23,24]. During the study period, the records of 372 patients were eligible for analysis, of which 351 conformed to all inclusion criteria, and 21 were removed due to the exclusion criteria (Figure 1).

### 2.2. Definition of PHN

The evaluation period was between 1 and 6 months after the initial outbreak of HZ. Clinically relevant PHN was defined as the presence of continuous pain that persists beyond 3 months postinfection, with a severity level of 3 or more on the numerical rating scale (NRS) [5,25]. Cases with an NRS score <3 or in which treatment was discontinued due to pain alleviation were categorized as non-PHN. The study focused on patient evaluation until 6 months post-HZ onset. Patients who registered an NRS score of 3 or greater at the end of this six-month evaluation were allocated to the PHN group.

### 2.3. Measurement of Psoas: Lumbar Vertebral Index (PLVI)

**Figure 2:**
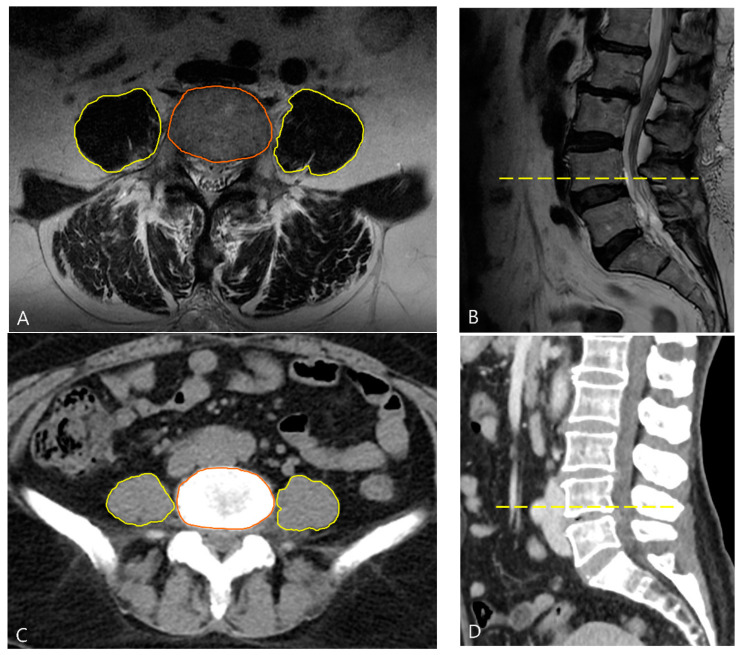
Representative cross-sectional (**A**) and sagittal (**B**) MRI images. Measurement of the right and left psoas muscle areas (outlined in yellow) and vertebral body area (outlined in orange) at the L4 level. Cross-sectional image was obtained at the level of the dashed line in (**B**). (**C**,**D**) Representative cross-sectional and sagittal abdominopelvic CT images, respectively. Measurement of the right and left psoas muscle areas (outlined in yellow) and vertebral body area (outlined in orange) at the L4 level. Cross-sectional image was obtained at the level of the dashed line in (**D**). CT, computed tomography; MRI, magnetic resonance imaging.

The CT images were reviewed by two anesthesiologists who were blinded to patient information in the picture archiving and communication system (PACS). To calculate the PLVI, we obtained measurements of the average area of the right and left psoas muscles (psoas muscle cross-sectional area [PCSA]) relative to the combined area of the lumbar vertebrae. Cross-sectional MRI or CT images at the level of the inferior endplate of the L4 vertebra were selected for analysis. The dimensions of the psoas muscles bilaterally and of the L4 vertebral body were manually determined using PACS software (Figure 2). The PLVI was calculated using the following formula: 

$\left([right\;PCSA\;({\mathrm{mm}}^{2})+left\;PCSA\;({\mathrm{mm}}^{2})]/2\right)/L4\;vertebral\;CSA\;({\mathrm{mm}}^{2})$ [16,18]. The median PLVI, segregated by sex, was computed, and the patients were divided into low- or high-PLVI groups.

### 2.4. Assessment of Frailty 

The electronic medical records of all participants were screened to calculate the modified frailty index (mFI), a metric of frailty. The mFI was adapted from the Canadian Study of Health and Aging Frailty Index [10] by aligning 70 variables into 11 categories of health deficits and comorbid conditions. The relevant components were extracted from each patient’s health record and classified across 11 comorbidities (detailed in Table S1), with the final score being the total number of these factors divided by 11 [15,26]. The patients were then stratified into not frail (including those considered pre-frail), with mFI scores ranging from 0 to 26, and frail, with mFI scores ≥ 0.27 [15,26]. For the logistic regression analysis aimed at predicting the occurrence of PHN, we formed 11 categorical groups based on the mFI scores, from 0 to 1 in a stepwise manner.

### 2.5. Outcome Measures

The primary outcome was the efficacy of the PLVI in predicting PHN in older patients, as evaluated using receiver operating characteristic (ROC) curves. The secondary outcome was to assess whether the mFI, a measure of frailty, can predict PHN. The level of pain reported by the patients at their first consultation was evaluated using the NRS. The study population did not include patients treated with anticonvulsants, except for gabapentin and pregabalin. Anticonvulsant dosages were normalized to equivalent doses of pregabalin [27,28] and opioid dosages were standardized using the morphine equivalent daily dose [29]. Interventional procedures, such as the stellate ganglion block, paravertebral block, selective nerve root block, and similar treatments, were quantified by the number of times they were performed, without distinguishing between the different anatomical locations or specific types of procedures.

### 2.6. Statistical Analysis

Continuous variable normality was examined using the Shapiro–Wilk test. The continuous variables are reported as the mean ± standard deviation if normally distributed, or as the median with interquartile range if not. The categorical variables are presented as counts (percentage). Differences between the two groups for continuous data were evaluated using the independent samples *t*-test for normally distributed data, or the Mann–Whitney U test for non-normally distributed data. For categorical data, we used the chi-square test or Fisher’s exact test as appropriate. ROC curve analysis determined whether patients with and without PHN could be distinguished based on the PLVI and mFI. The data are presented as the area under the curve (AUC) with 95% confidence intervals (CIs). The PLVI cutoffs for PHN and mFI were identified using Youden’s index, alongside their respective sensitivity and specificity values. The model fit was evaluated using the Hosmer–Lemeshow test and AUC values. The association between the PLVI and mFI, and the risk factors for PHN development were examined using binary logistic regression analysis. Predictors of PHN were further analyzed using multivariate logistic regression with a backward elimination approach, and the results are presented as odds ratios (ORs) with 95% CIs. Statistical analysis was conducted using the MedCalc Statistical Software Version 20.1.4 2020 (MedCalc Software bv, Ostend, Belgium) and IBM SPSS Statistics (version 22.0; IBM Corp., Armonk, NY, USA). A *p*-value of <0.05 was considered to indicate statistical significance, employing two-tailed *p*-values for testing.

## 3. Results

Significant differences were observed between the postherpetic neuralgia (PHN) and non-PHN groups in terms of the presence of comorbidities (diabetes mellitus, chronic heart failure, a cerebrovascular accident with sequelae, or pulmonary disease), dependent functional status, number of selective nerve root blocks, numerical rating scale (NRS) scoring, and pain duration. There were no significant differences in other factors between the two groups (Table 1).

**Figure 3:**
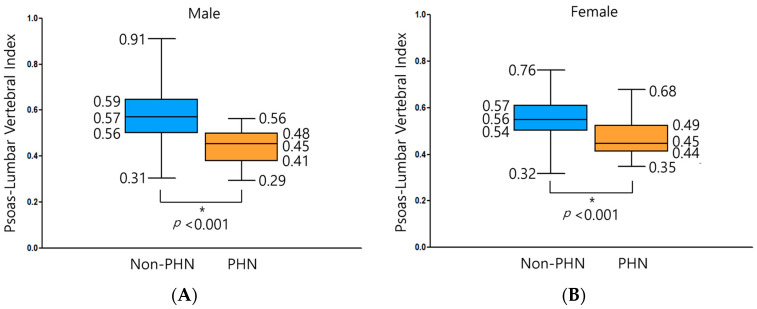
Box plots showing the distribution of the values for the psoas–lumbar vertebral index (PLVI) in the non-PHN and PHN groups in men (**A**) and women (**B**). Top-to-bottom data next to each box plot indicate the maximum, upper quartile, median, lower quartile, and minimum values of PLVI. PHN, postherpetic neuralgia. * *p* values were calculated using *t*-test.

In male patients, the psoas: lumbar vertebral index (PLVI) was 0.57 ± 0.10 in the non-PHN group and 0.45 ± 0.08 in the PHN group (*p* < 0.001). For female patients, the PLVI was measured at 0.56 ± 0.08 and 0.45 ± 0.08 in the non-PHN and PHN group, respectively (*p* < 0.001) (Figure 3). Cross-analysis between the two groups revealed that 79.7% of the patients in the PHN group had a low PLVI, and 42.2% of the patients in the same group were frail (modified frailty index (mFI) ≥ 0.27); both values were significantly different from those in the non-PHN group (Table 2).

**Figure 4:**
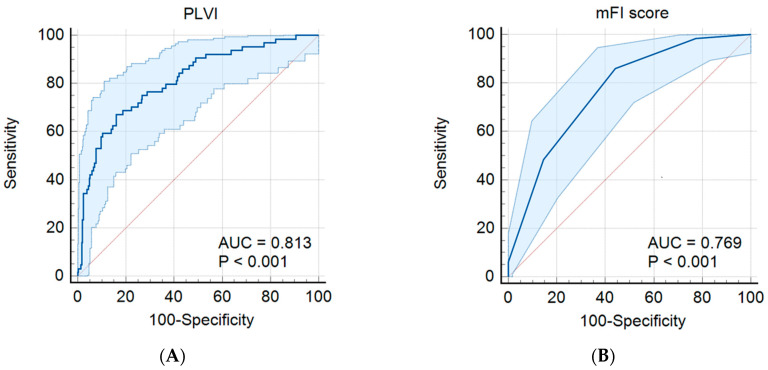
Receiver-operating characteristic curves for predicting PHN using (**A**) PLVI and (**B**) mFI score. AUC; area under the curve; PHN, postherpetic neuralgia; PLVI, psoas: lumbar vertebral index; mFI, modified frailty index.

The receiver operating characteristic (ROC) curves (Figure 4) showed that the area under the curve (AUC) values for PLVI and mFI were 0.81 (95% confidence interval (CI), 0.77–0.85; *p* < 0.001) and 0.77 (95% CI, 0.72–0.81; *p* < 0.001), respectively. The optimal cutoff values of PLVI and mFI for predicting PHN were 0.048 (67.2% sensitivity and 84.0% specificity) and 0.09 (85.9% sensitivity and 55.7% specificity), respectively.

Univariate analysis showed that NRS score, pain duration, PLVI category (cutoff), mFI variables (categorical), comorbidities (diabetes mellitus, chronic heart failure, a cerebrovascular accident with sequelae, and pulmonary disease), and dependent functional status were significantly associated with PHN. In particular, the emergence of PHN was strongly linked to a low PLVI (odds ratio (OR) 5.62; 95% CI, 2.93–10.793; *p* < 0.001), and the number of mFI variables (OR 5.48; 95% CI, 3.04–9.88; *p* < 0.001). Multivariate logistic regression analysis revealed that the NRS scores (OR 1.30; 95% CI, 1.07–1.58; *p* = 0.008), a low PLVI (OR 3.27; 95% CI, 1.52–7.00; *p* = 0.002), and the number of mFI variables (OR 2.46; 95% CI, 1.21–4.50; *p* = 0.013) were significant independent predictors of PHN (Table 3).

## 4. Discussion

In this retrospective analysis, we investigated the correlation between psoas: lumbar vertebral index (PLVI) categories and the occurrence of postherpetic neuralgia (PHN), along with its association with frailty scores. Receiver operating characteristic (ROC) curve analysis revealed area under the curve (AUC) values of 0.813 for the PLVI and 0.769 for the modified frailty index (mFI), showcasing a superior predictive capability of PLVI over mFI. Moreover, through multiple regression analysis, PLVI was significantly linked to PHN development, with a higher odds ratio than the mFI, highlighting an association between a low PLVI in the older population and PHN emergence.

Herpes zoster (HZ) can precipitate zoster-associated pain, which includes both acute pain and the chronic condition known as PHN. Although not life-threatening, PHN significantly undermines the daily functioning, physical capabilities, and overall life satisfaction of those affected. The levels of the zinc finger antiviral protein arise due to viral harm and heightened sensitivity in the sensory neurons of the affected segments, leading to damage to both peripheral and central nerves, inflammatory reactions, immune disturbances, and neuronal degradation within the spinal ganglia [4]. Between 8% and 24% of individuals with HZ go on to experience PHN, resulting in a notable deterioration in their quality of life across various dimensions, including physical, psychological, functional, and social [4,19]. The underlying mechanisms of PHN development are yet to be fully elucidated despite extensive research [3,19]. Several risk factors have been linked to PHN, including diminished cell-mediated immunity, older age, female sex, presence of a prodromal stage, intense rash severity, acute severe pain, lack of adequate nutrition, and psychosocial stressors [1,6,7]. People aged 60 years and older are at an increased risk of developing both HZ and PHN, with evidence suggesting that PHN prevalence escalates with age [5,30]. The continual year-on-year rise in PHN cases and associated healthcare costs are thought to be tied to the increasing older population and higher life expectancy [4,5,6,8].

Numerous studies have confirmed the significance of frailty in predicting disease progression and mortality among older populations, confirming its value as a dependable metric [9,10,14,21,26]. Practical applications of frailty assessments in clinical settings are hindered by their dependence on questionnaire-based evaluations [10]. This challenge becomes more acute in older patients with cognitive deficits, potentially limiting their engagement in the assessment procedure. Sarcopenia is often identified as an early-stage condition or a physical indicator of frailty and is closely linked to an elevated risk of negative health events such as falls, bone fractures, and physical impairments [9,14]. The assessment of central sarcopenia by measuring the sizes of key muscle groups, notably the psoas muscle, offers valuable insights. Given the influence of sarcopenia, evaluation of the psoas muscle area serves as an indicator of an individual’s capacity for active daily living, and provides a dynamic snapshot of a patient’s physical condition [18,20].

Several studies have proposed psoas muscle metrics as an alternative to traditional frailty evaluations in older adults. Recent research has suggested that the size of the psoas muscle is a predictive marker for patient outcomes. Its prognostic significance has been highlighted in the literature in relation to hip fracture outcomes [15,16], and muscle size has been recognized as a predictive factor for recovery following cardiac procedures [20,21]. In addition to its role in predicting surgical outcomes, the measurement of psoas size has been recognized as a valuable prognostic tool across a spectrum of internal medical conditions in elderly patients. Ebbeling et al. [18] reported that the PLVI showed no association with mortality but was independently and negatively associated with morbidity in a cohort of 180 elderly trauma patients. They suggested that the PLVI enabled rapid and straightforward prediction of increased complication risks in elderly trauma patients. Also, Okada et al. [22] evaluated 255 sepsis patients with a median age of 76 who were admitted to the intensive care unit. Using computed tomography (CT) imaging, they measured the psoas index and categorized patients into “Middle” and “Sarcopenic” groups. The study reported an association between sarcopenic findings in the psoas muscle and 90-day mortality. Nevertheless, the cutoff values for the total psoas area may need an adjustment for the patient’s body size, suggesting a requirement for calibration to accurately reflect body stature. Given these considerations, recent studies have shown a preference for the PLVI rather than relying solely on total psoas area, aiming to offer a more individualized assessment that accounts for a patient’s body habitus and height [16,18]. Consequently, in this study, we opted to calculate the PLVI instead of the total psoas area.

Age-related decline in immune function, known as immunosenescence, is a well-established phenomenon that encompasses heightened vulnerability to infections, diminished efficacy of vaccinations in older adults, and an elevated likelihood of developing chronic inflammatory conditions [9,12]. A key aspect of immunosenescence is inflammaging, defined as the age-associated increase in chronic, low-grade systemic inflammation [9,11]. Inflammation has been linked to anorexia and the breakdown of both skeletal muscle and fat, potentially leading to nutritional deficits, muscle weakening, and body weight loss, which typify frailty [31,32]. Consequently, mild levels of inflammation, such as inflammaging, might not directly cause a decrease in muscle mass or strength; however, they could still play a role in the onset of sarcopenia by affecting metabolic integrity [9,33]. A notable number of epidemiological studies have established a direct correlation between persistently high levels of serum IL-6 and frailty, indicating that increased IL-6 levels are a harbinger of the development of sarcopenia [31,32]. IL-6 operates through the ubiquitin–proteasome pathway responsible for muscle degradation, with elevated systemic IL-6 levels being linked to more active ubiquitin protein and proteasome processes. Furthermore, expression of IL-6 can lead to insulin resistance, which effectively dampens muscle protein synthesis [34,35]. 

Specifically, IL-6, which is closely linked to inflammaging and sarcopenia, has been identified in multiple studies as being associated with PHN [36,37,38]. Zhu et al. [36] reported that IL-6 concentrations in patients with PHN were elevated compared to those in patients with HZ who did not develop PHN. A direct relationship was observed between IL-6 levels and pain intensity, recorded using the visual analog scale (VAS) in individuals with PHN, showing an association with a high risk for PHN. Individuals with HZ who went on to develop PHN experienced more severe pain and had higher serum IL-6 levels during the acute phase of the disease than those who did not develop PHN. Therefore, the inflammatory response, especially that involving IL-6, during the acute phase of HZ is implicated in hyperalgesia and the onset of PHN. Numerous studies have described PHN as a form of neuropathic pain that is potentially linked to different types of nervous system impairment [37,39]. The production of IL-6 by macrophages, mast cells, lymphocytes, neurons, and glial cells is typically minimal and aids in the normal growth and repair processes of the nervous system, whereas elevated IL-6 levels can lead to nervous system damage [40,41]. Thus, among cytokines linked to aging and inflammaging, which are implicated in sarcopenia and frailty, IL-6 is a critical contributor to PHN. While a concrete mechanism that establishes a direct link between these conditions remains to be definitively established, it is reasonable to consider their interrelationship.

ROC curve analysis indicated the superior prognostic capability of the PLVI over the mFI in PHN. While the initial univariate conditional logistic regression identified nine potential predictors for PHN, the subsequent conditional multivariate logistic regression analysis retained significance for only three predictors: the numerical rating scale (NRS), the PLVI, and the mFI. Although frailty, which reflects the general health status of older individuals, is associated with PHN, the results of this study suggest that the PLVI assessment more accurately reflects the prognostic likelihood of PHN. In this study, a diminished PLVI was linked to lower levels of physical activity, which in turn correlated with a heightened risk of PHN development. Consequently, a lower paraspinal muscle area, when adjusted for body surface area, significantly correlated with the risk of PHN development. It has been documented that impaired physical function influences the emergence of PHN after HZ infection [1,2,3,4]. Furthermore, Chae et al. [4] observed that among older individuals with spinal conditions, the occurrence of HZ at the same spinal level as the affected neural segment was associated with more severe zoster-related discomfort. Bouhassira et al. [2] reported that a diminished physical component summary score served as an independent predictive factor for PHN. Similarly, Drolet et al. [1] found that advanced age and existing issues with mobility prior to contracting HZ significantly increased the likelihood of developing PHN. In their multivariate regression analysis, Kawai et al. [3] identified that factors such as older age (between 60 and 69 years compared to between 50 and 59 years), pain severity at rash onset, employment status, experiencing mobility issues at the start of the evaluation period, and pain affecting interpersonal relationships were notably linked to an increased risk of PHN development. These findings collectively suggest that reduced physical activity, indicated by a low PLVI, may be a crucial risk factor for the onset of PHN. The PLVI offers a quantitatively objective approach and is easier to obtain than traditional methods for frailty assessment. This measure greatly reduces the necessity for additional diagnostic tests, as PLVI metrics can be readily determined from cross-sectional imaging modalities, including lumbar CT, magnetic resonance imaging (MRI), or abdominal–pelvic CT scans, which are routinely performed for overall health evaluations or investigations of lower back pain. Additionally, deriving PLVI values does not require the patient’s active involvement or extensive medical history, which is notably advantageous in older patients with cognitive impairments.

Assessing the risk of PHN development in patients with HZ is of paramount importance. Numerous studies have shown that early adoption of supplemental therapies can significantly reduce the incidence of PHN [3,19]. Prompt commencement of drug therapies or early implementation of supplementary interventional techniques, such as sympathetic ganglion block or nerve block, can substantially reduce the occurrence of PHN [19]. A restricted inflammatory reaction during the acute phase of HZ infection may result in inadequate containment of the virus and more significant damage within the affected dermatome, leading to prolonged neuralgia [3,19]. Assessing the degree of risk through metrics such as the PLVI and mFI, and providing early, proactive treatment for those at elevated risk can aid in preventing progression to PHN. Such preventive measures are not only expected to enhance the quality of life of older individuals afflicted with shingles by reducing pain, but also to yield substantial social and economic benefits for the society.

This study had several limitations. First, it was a single-center study that encompassed a relatively small patient cohort, predominantly of South Korean ethnicity. Expanding to a multicenter format with a larger and more diverse participant base may be beneficial. Second, imaging records were not available for all patients with shingles, which limited our study to those with accessible imaging data. In a prospective study setup, imaging-related expenses would unavoidably increase. Third, a universally accepted cutoff value for the PLVI as an indicator of central sarcopenia in individuals aged 60 years and older is yet to be established. For the purposes of this study, the individuals were categorized into two groups based on the median PLVI values, assuming the sarcopenia was present in the group with lower values. Fourth, frailty was exclusively assessed using the mFI, which was derived retrospectively from identifiable comorbidities or deficits and not through questionnaire-based evaluations. Lastly, manual demarcation of the muscle, which could inadvertently include perimuscular fat in the measured area, posed an additional limitation. To counteract potential discrepancies and enhance accuracy, PLVI measurements were conducted by two board-certified anesthesiologists.

## 5. Conclusions

This study identified a significant association between reduced PLVI and the incidence of PHN in individuals aged 60 years and older affected by HZ. Despite the study’s limited sample size, single institution setting, and retrospective design constraining the generalizability and ability to establish causality, our findings highlight the potential of the PLVI, in conjunction with frailty scores, as a predictive tool for assessing PHN risk in this population. To validate these findings and further understand the underlying mechanisms, future research should involve larger, multi-center cohorts and longitudinal studies.

## Figures and Tables

**Table 1:** Demographic and baseline characteristics of the participants.

	Non-PHN (n = 287)	PHN (n = 64)	*p*-Value
Age, year	72.06 ± 8.206	73.98 ± 8.638	0.094 ^a^
Sex, female	59.2% (170/287)	67.2% (43/64)	0.260 ^b^
BMI, kg/m^2^	23.551 ± 3.156	22.740 ± 2.466	0.055 ^a^
Hypertension	35.2% (101/287)	46.9% (30/64)	0.088 ^b^
DM	12.9% (37/287)	23.4% (15/64)	**0.035** ^b^
CHF	12.5% (36/287)	23.4% (15/64)	**0.031** ^b^
Cardiac intervention or angina	8.7% (25/286)	15.6% (10/64)	0.107 ^b^
MI	8.7% (25/286)	15.6% (10/64)	0.107 ^b^
TIA or CVA	15.7% (45/287)	25.0% (16/64)	0.099 ^b^
CVA with sequelae	7.0% (20/286)	17.5% (11/64)	**0.013** ^b^
Dependent functional status	4.9% (14/286)	14.1% (9/64)	**0.012** ^b^
Peripheral disease	10.8% (31/287)	18.8% (12/64)	0.092 ^b^
Pulmonary disease	9.4% (27/286)	20.3% (13/64)	**0.018** ^b^
Impaired sensory	10.5% (30/286)	18.8% (12/64)	0.086 ^b^
Number of SNRBs	2.86 (2.00)	3.23 (3.00)	**0.021** ^c^
Dermatome affected			0.997 ^d^
Facial	34 (11.8%)	8 (12.5%)	
Cervical	62 (21.6%)	13 (20.3%)	
Thoracic	123 (42.9%)	27 (42.2%)	
Lumbar	58 (20.2%)	14 (21.9%)	
Sacral	10 (3.5%)	2 (16.7%)	
NRS	6.66 ± 1.626	7.50 ± 1.345	**<0.001** ^a^
Pain duration	20.30 ± 14.189	25.61 ± 18.268	**0.011** ^a^
MME	17.719 ± 17.152	18.207 ± 16.583	0.836 ^a^
Anticonvulsant	195.96 ± 85.704	201.56 ± 90.325	0.640 ^a^

Values are presented as the mean ± standard deviation, median (interquartile range), or number of patients (%). BMI, body mass index; CHF, chronic heart failure; CVA, cerebrovascular accident; DM, diabetes mellitus; HTN, hypertension; MI, myocardial infarction; MME, morphine milligram equivalents; NRS, numeral rating scale; PHN, postherpetic neuralgia; SNRBs, selective nerve root blocks; TIA, transient ischemic attack. *p*-values < 0.05 are in bold. ^a^ *t*-test; ^b^ Chi-square test; ^c^ Mann–Whitney U test; ^d^ Fisher’s exact test.

**Table 2:** Patient groups according to PLVI category and frailty assessment.

	No-PHN (n = 287)	PHN (n = 64)	*p*-Value
PLVI			
High PLVI	58.9 (169/287)	20.3 (13/64)	**<0.001**
Low PLVI	41.1 (118/287)	79.7 (51/64)	
Frailty			
Not-frail (mFI < 0.27)	85.4% (245/287)	51.6% (33/64)	**<0.001**
Frail (mFI ≥ 0.27)	14.6% (42/287)	48.4% (31/64)	

PHN, postherpetic neuralgia; PLVI, psoas: lumbar vertebral index; mFI, modified frailty index; *p*-values < 0.05 are in bold. *p*-values were calculated using Chi-square test.

**Table 3:** Unadjusted and adjusted odds ratios for predicting PHN using binary logistic regression.

Variables	Unadjusted Analysis		Adjusted Analysis	
	Odds Ratio (95% CI)	*p*-Value	Odds Ratio (95% CI)	*p*-Value
Age (for 1 year increase)	1.027 (0.995, 1.060)	0.095		
Sex (female)	1.409 (0.795, 2.498)	0.240		
NRS	1.409 (1.175, 1.690)	**<0.001**	1.300 (1.071, 1.579)	**0.008**
Number of SNRBs	1.093 (0.961, 1.242)	0.176		
Pain duration	1.021 (1.005, 1.039)	**0.012**		
Low PLVI	5.619 (2.925, 10.793)	**<0.001**	3.265 (1.523, 7.001)	**0.002**
mFI variables (categorical)	5.480 (3.039, 9.880)	**<0.001**	2.455 (1.211, 4.976)	**0.013**
HTN	1.625 (0.940, 2.809)	0.082		
DM	2.068 (1.055, 4.057)	**0.034**		
CHF	2.134 (1.086, 4.195)	**0.028**		
Cardiac intervention or angina	1.941 (0.881, 4.275)	0.100		
MI	1.941 (0.881, 4.275)	0.100		
TIA or CVA	1.793 (0.937, 3.431)	0.078		
CVA with sequelae	2.824 (1.277, 6.244)	**0.010**		
Dependent functional status	3.191 (1.316, 7.740)	**0.010**		
Peripheral disease	1.906 (0.918, 3.955)	0.083		
Pulmonary disease	2.455 (1.187, 5.076)	**0.015**		
Sensory impairment	1.977 (0.950, 4.114)	0.068		

CHF, chronic heart failure; CI, confidence interval; CVA, cerebrovascular accident; DM, diabetes mellitus; HTN, hypertension; mFI, modified frailty index; MI, myocardial infarction; MME, morphine milligram equivalents; NRS, numeral rating scale; PHN, postherpetic neuralgia; PLVI, psoas: lumbar vertebral index; SNRBs, selective nerve root blocks; TIA, transient ischemic attack; *p*-values < 0.05 are in bold. Adjusted odds ratios are derived from a multivariable logistic regression adjusting for variables with a *p*-value < 0.05 in the unadjusted analysis: NRS, pain duration, low PLVI, mFI variables (categorical), DM, CHF, CVA with sequelae, dependent functional status, and pulmonary disease.

## Data Availability

The datasets are available upon reasonable request to the corresponding author.

## Supplementary Materials

The following supporting information can be downloaded at https://www.mdpi.com/article/10.3390/jcm13113100/s1: Table S1: Factors of modified frailty index.
