# Supplementary material for: Association of Psoas: Lumbar Vertebral Index (PLVI) with Postherpetic Neuralgia in Patients Aged 60 and Older with Herpes Zoster
Source: J Clin Med. 2024 May 25;13(11):3100. doi: 10.3390/jcm13113100 (PMC11172933; doi:10.3390/jcm13113100)
Supplement: Supplementary file 1 [file jcm-13-03100-s001.zip › jcm-2995507-supplementary.pdf]

Supplementary Table S1. Factors of modified frailty index

History of hypertension requiring medication
History of diabetes mellitus[a]
History of congestive heart failure
History of either prior PCI, PCS at any time, or angina within 30 days
History of myocardial infarction within 6 months
History of either transient ischemic attack or cerebrovascular accident
History of a cerebrovascular accident with neurological deficit
Non-independent functional status (partially or totally dependent activities of daily living)
History of either peripheral vascular disease or rest pain
History of either COPD exacerbation or pneumonia within 30 days
History of impaired sensorium

COPD, chronic obstructive pulmonary disease; PCI, percutaneous coronary intervention; PCS, prior cardiac surgery. [a] diabetes controlled by diet alone, treated with oral anti-hyperglycemic therapy or with insulin.
